# Theoretical and Experimental Electrostatic Potential around the *m*-Nitrophenol Molecule

**DOI:** 10.3390/molecules20034042

**Published:** 2015-03-03

**Authors:** Mokhtaria Drissi, Nadia Benhalima, Youcef Megrouss, Rahmani Rachida, Abdelkader Chouaih, Fodil Hamzaoui

**Affiliations:** Laboratoire LTPS, Faculté des Sciences et de la Technologie, Université de Mostaganem, 27000-Mostaganem, Algeria; E-Mails: mokadrissi@gmail.com (M.D.); nadia1lotus@yahoo.fr (N.B.); youmeg@hotmail.fr (Y.M.); rahmani_ra63@yahoo.fr (R.R.); aek_chouaih@yahoo.fr (A.C.)

**Keywords:** electron charge density, *m*-nitrophenol, nonlinear optical compound (NLO), electrostatic potential, optimized geometry, HOMO-LUMO

## Abstract

This work concerns a comparison of experimental and theoretical results of the electron charge density distribution and the electrostatic potential around the *m*-nitrophenol molecule (m-NPH) known for its interesting physical characteristics. The molecular experimental results have been obtained from a high-resolution X-ray diffraction study. Theoretical investigations were performed using the Density Functional Theory at B3LYP level of theory at 6-31G* in the Gaussian program. The multipolar model of Hansen and Coppens was used for the experimental electron charge density distribution around the molecule, while we used the DFT methods for the theoretical calculations. The electron charge density obtained in both methods allowed us to find out different molecular properties such us the electrostatic potential and the dipole moment, which were finally subject to a comparison leading to a good match obtained between both methods. The intramolecular charge transfer has also been confirmed by an HOMO-LUMO analysis.

## 1. Introduction

**Figure 1:**
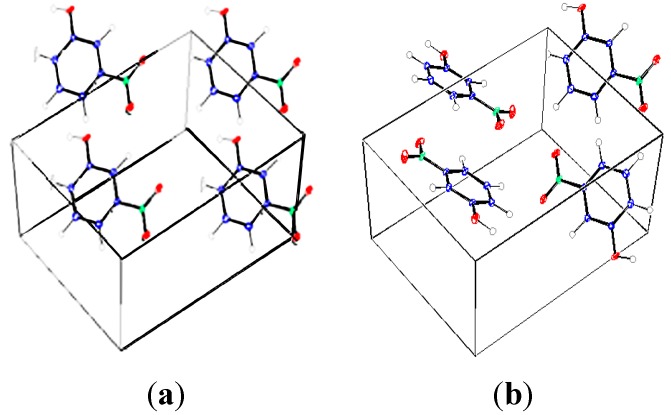
Polymorphic forms of *m*-nitrophenol, (**a**) orthorhombic, (**b**) monoclinic.

*m*-Nitrophenol (m-NPH) occurs in two polymorphic forms: orthorhombic (P2_1_2_1_2_1_) and monoclinic (P2_1_/n) (see Figure 1). We are going to concentrate on the monoclinic form as the first form has already been the subject of a preceding report [1]. The main purpose of our work is to establish the electrostatic potential around the molecule through the determination of the electron charge density. This electrostatic potential will help us to describe and understand the inter- and intramolecular interactions (charge transfer) in the crystal. The presented electrostatic potential can be a starting point for the estimation of crystal energy cohesion in order to get more information about the existence of the polymorphism in the compound *m*-nitrophenol [2,3,4].

We have previously published an article about the high resolution X-ray diffraction and crystallographic study with a thermal motion analysis of the compound m-NPH [5]. The lasting metastability of the monoclinic form of *m*-nitrophenol originates probably from interactions within the centrosymmetric dimers of overlapping molecules. The attractive interactions within centrosymmetric dimers of overlapping molecules are as strong as interactions between hydrogen-bonded molecules [6,7]. We also presented the molecular dipole moment based on spherical model refinement [8]. In the present work, we based our study on the multipolar model of Coppens–Hansen where the non-spherical aspect of the atoms was taken into account. The multipole model represents an extrapolation to infinite resolution from a finite set of experimental data [9]. This last refinement gave us an accurate picture of the electronic charge density distribution in the compound m-NPH. 

The theoretical part adds to our experimental work by using *ab-initio* calculations through providing a comparison of the molecular electrostatic properties such as the dipole moment, the electron density maps and the electrostatic potential with the experimental data.

## 2. Experimental Details

The accurate electron density distribution and the electrostatic potential around the molecule (m-NPH) have been calculated from a high-resolution X-ray diffraction study [5]. A summarized table of the X-ray experiment details of the crystallographic data is given in Table 1.

**Table 1:** Experimental details.

Crystal Data	**
Chemical formula	C_6_H_5_NO_3_
Chemical formula weight	139.11
Cell setting	Monoclinic
Space group	P2_1_/*n*
*a* (Å)	11.026 (4)
*b* (Å)	6.736 (1)
*c* (Å)	8.119 (21)
β (°)	97.73 (2)
*V* (Å^3^)	597.50
Z	4
Radiation type	Mo *K*α
Temperature (K)	122 (1)
No. of measured reflections	3148

The Hansen-Coppens multipole formalism [10], as implemented in the MOPRO least squares program [11] for multipole refinement, was used for both observed and theoretical structure factor fitting. This package is based on program of least square method using non spherical electron distribution around the atoms [12,13].

The rigid pseudo-atom model Hansen-Coppens is commonly used in analysis of the charge density distribution. The electron density $\rho \left(\vec{r}\right)$ in the crystal is described by a sum of so-called aspherical pseudo-atoms with nuclear positions $\vec{r_{k}}$:

(1) $$\rho \left(\vec{r}\right)=\sum_{k}\rho_{k}\left(\vec{r}-\vec{r_{k}}-u\right)*t_{k}\left(u\right)$$

where $t_{k}\left(u\right)$ is a Gaussian thermal-displacement distribution and * indicates a convolution product. The different atomic densities are described as a series expansion in real spherical harmonic functions *Y_lm_* up to order four:

(2) $$\rho_{atom}\left(\vec{r}\right)=\rho_{c}\left(\vec{r}\right)+P_{v}K^{\prime 3}\rho_{v}\left(K\prime \vec{r}\right)+\sum_{l=0}^{4}\sum_{m=-l}^{+l}K\prime \prime R_{l}\left(K\prime \prime \vec{r}\right)P_{lm}Y_{lm}\left(\frac{\vec{r}}{r}\right)$$

In Equation (2), $\rho_{c}$ and $\rho_{v}$ are spherically averaged Hartree-Fock core and valence densities, with $\rho_{v}$ normalized to one electron, $Y_{lm}$ are multipolar spherical harmonic angular functions in real form and $R_{l}$ are Slater–type radial functions.

Two charge-density variables, $P_{v}$ and $P_{lm}$, the population parameters, and K′ and K′′ parameters which allows expansion and contraction of the valence shell, are added to the conventional structural analysis parameters [14]. The population parameters $P_{v}$ and $P_{lm}$ are floated along with K′ and K′′ during the refinement. To reduce the number of variables, atoms having the same environment were assumed to have the same electron charge deformation: All hydrogen atoms were assumed to be equivalent and are described by the same Slater radial function. Also, the two oxygen atoms are assumed to have the same local symmetry.

The least-square refinements method allowed us to the accurately get the net atomic charge, the molecular dipole moment and the electrostatic potential around the molecule. We have also described the electron density distribution in the crystal form.

## 3. Computational Details

The theoretical calculations were performedusingthe Density Functional Theory at B3LYP (Becke’s three parameter hybrid functional using the correlation functional of Lee, Yang, and Parr, which includes both local and non-local terms correlation functional) methods at 6-31G* level [15]. To perform this computational work, we used the Gaussian 09 program package [16]. The Highest Occupied Molecular Orbital (HOMO)-Lowest Unoccupied Molecular Orbital (LUMO) analysis has been carried out to explain the charge transfer place within the molecule. The chemical hardness and chemical potential are also calculated using the HOMO and LUMO. The visualization of the electron charge and the electrostatic potential of the molecule were obtained using the Molden program [17].

## 4. Results and Discussion

### 4.1. Optimization of Geometrical Parameters

Geometry optimization is a name for the procedure that attempts to find the configuration of minimum energy of the molecule. The procedure calculates the wave function and the energy at a starting geometry and then proceeds to search a new geometry of a lower energy.

**Figure 2:**
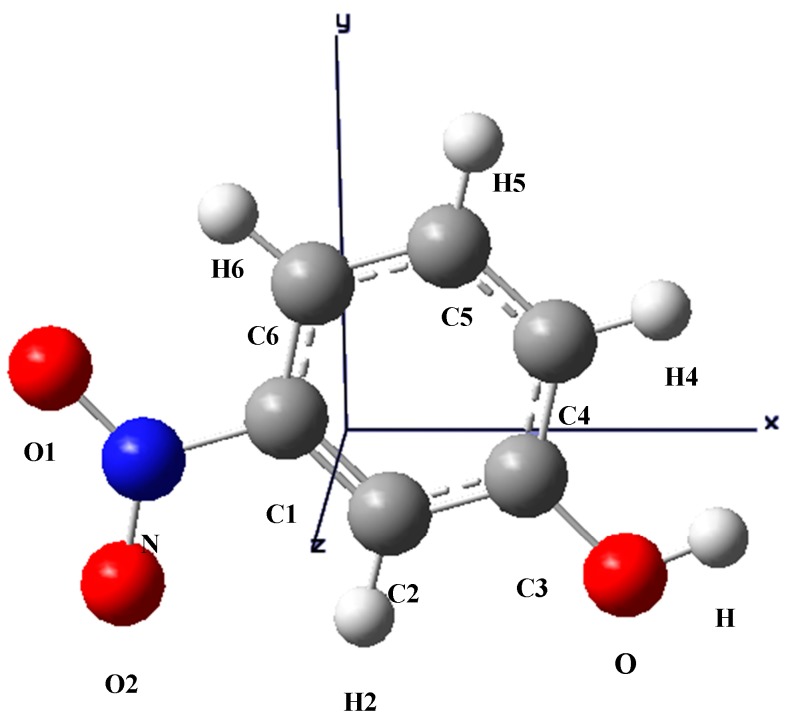
The optimized structure of m-NPH based on DFT B3LYP/6-1G* basis set.

The optimized structure of the title compound is shown in Figure 2. The calculated structure parameters (bond lengths, bond angles and torsion angles) were listed in Table 2, Table 3 and Table 4 where it can be seen that all the calculated parameters are in line with the X-ray results. In summary, the optimized bond lengths and bond angles obtained using the DFT method are in good agreement with the corresponding X-ray structural parameters. The calculated geometric parameters represent a good approximation and can provide a starting point to calculate other parameters, such as vibrational wavenumbers.

**Table 2:** Selected bond distances (Å) by X-ray and theoretical calculations (B3LYP/6-31G*).

Atom 1	Atom 2	Distance (Å)	
		X-ray	B3LYP/6-31G*
C1	C6	1.410	1.433
C1	C2	1.396	1.384
C2	C3	1.402	1.396
C3	C4	1.411	1.392
C4	C5	1.396	1.391
C6	C5	1.400	1.412
O	C3	1.365	1.380
C1	N	1.474	1.468
O1	N	1.244	1.281
O2	N	1.243	1.283
O	H	1.030	0.992
H6	C6	1.089	1.084
H2	C2	1.085	1.078
H4	C4	1.078	1.069
H5	C5	1.085	1.082

**Table 3:** Selected bond angles (°) by X-ray and theoretical calculations (B3LYP/6-31G*).

Atom 1	Atom 2	Atom 3	Angle (°)	
			X-ray	B3LYP/6-31G*
C6	C1	N	118.90	118.46
C6	C1	C2	124.11	122.55
N	C1	C2	116.99	117.37
H2	C2	C3	122.50	119.45
H2	C2	C1	120.51	120.82
C3	C2	C1	118.99	119.10
C4	C3	O	123.31	122.72
H4	C4	C3	120.31	119.88
H4	C4	C5	118.98	118.94
C3	C4	C5	120.71	118.06
H5	C5	C6	119.20	119.87
H5	C5	C4	120.66	119.74
C6	C5	C4	120.14	119.28
H6	C6	C5	119.39	120.13
H6	C6	C1	123.00	120.91
C5	C6	C1	117.60	119.28
H	O	C3	109.00	110.55
O2	N	O1	123.70	122.07
O2	N	C1	119.09	117.04
O1	N	C1	117.20	117.03

**Table 4:** Torsion angles (°) by X-ray and theoretical calculations (B3LYP/6-31G*).

Atom1	Atom 2	Atom 3	Atom 4	Angle (°)	
				X-ray	B3LYP/6-31G*
C4	C3	C2	C1	−1.07	0.003
C5	C6	C1	C2	0.92	−0.015
C4	C3	C2	C1	0.08	0.011
H2	C2	C3	C4	178.76	179.99
H6	C6	C1	C2	−179.28	−180.00
H5	C5	C6	C1	178.63	179.98
H4	C4	C3	C2	−179.68	−179.99
O	C3	C4	C5	−179.68	−179.97
H	O	C3	C4	−6.70	−179.98
N	C1	C2	C3	−179.84	0.011
O1	N	C1	C2	179.21	179.84
O2	N	C1	C2	0.39	0.020

### 4.2. Electron Density Maps 

**Figure 3:**
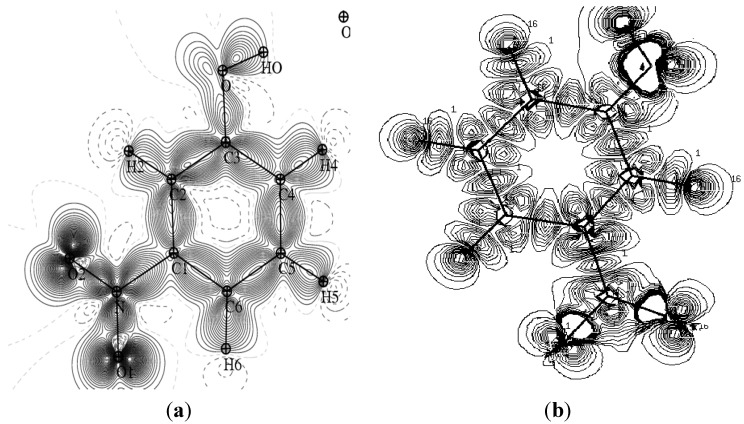
Comparison of the static and theoretical density maps of m-NPH. (**a**) Static density map. (**b**) Theoretical electron density map.

Figure 3 provides a comparison of the experimental static charge density of the molecule, obtained by convolution of the thermal motion from the charge density on the different atoms in the mean molecular plane, with the theoretical charge density, determined from a wave function for a pseudo atoms from an *ab initio* calculation performed with a Gaussian basis setusing the Density Functional Theory at B3LYP level of theory at 6-31G*. As it can be seen, the two maps show reasonable agreement. These maps confirm the high quality of the data sets and the efficiency of the formalism of data processing as proposed by Blessing [18].

### 4.3. Net Atomic Charges

**Figure 4:**
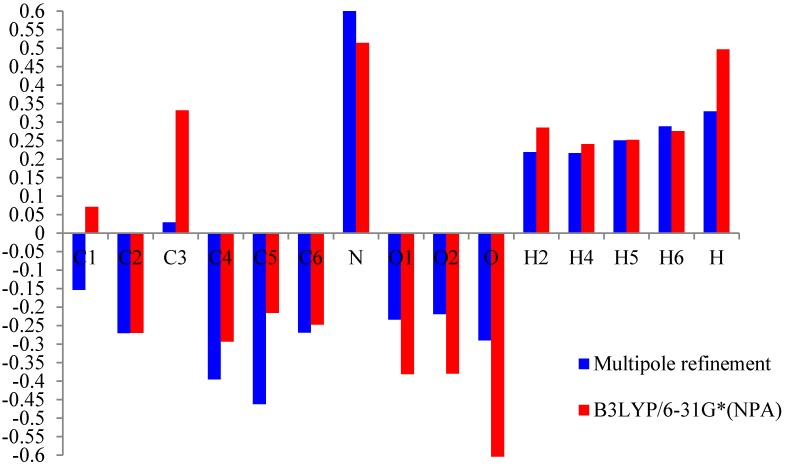
Histogram of the value of the net atomic charge in both methods multipolar refinement and B3LYP of *m*-nitrophenol.

Thevalence and mulitipolarpopulationceofficients were used to estimate the partial charges on the different atoms and the molecular dipole moment following the procedure described by Hansen and Coppens [8]. The experimantal net atomic charges have been previously published in an article about the high resolution X-ray diffraction and crystallographic study of m-NPH [5]. These values arecompared to the natural population analysis (NPA) charges derived from the *ab initio* calculations using B3LYP with the 6-31G* basis set (see Table 5, Figure 4). All the methods are in agreement for the evaluation of the positive sign of the net charges on the H and N atoms and the negative net charges on the O atoms. 

**Table 5:** Atomiccharge of *m*-nitrophenol.

Atom	Multipolar Refinement	B3LYP/6-31G*
C1	−0.1536	0.07099
C2	−0.2703	−0.26992
C3	0.0288	0.33201
C4	−0.3958	−0.29354
C5	−0.4621	−0.21591
C6	−0.2689	−0.24740
N	0.6466	0.51462
O1	−0.2337	−0.38131
O2	−0.2189	−0.38013
O	−0.2901	−0.68228
H2	0.2187	0.28515
H4	0.2165	0.24054
H5	0.2508	0.25207
H6	0.2885	0.27564
H	0.3289	0.49644

### 4.4. Molecular Moments

From the knowledge of the density function one can derive some important physical properties of the molecules such as the surrounding electrostatic field gradient, and the different electrostatic moments of the charge distribution [14]. A property associated to the average value of a quantum observable 〈*O*〉 is linked to the density function as given by the general equation (3), *V* is the molecular volume:

(3) $$\langle O\rangle =\int_{V}\hat{O}\left(\vec{r}\right)\rho \left(\vec{r}\right)d\vec{r}$$

**Figure 5:**
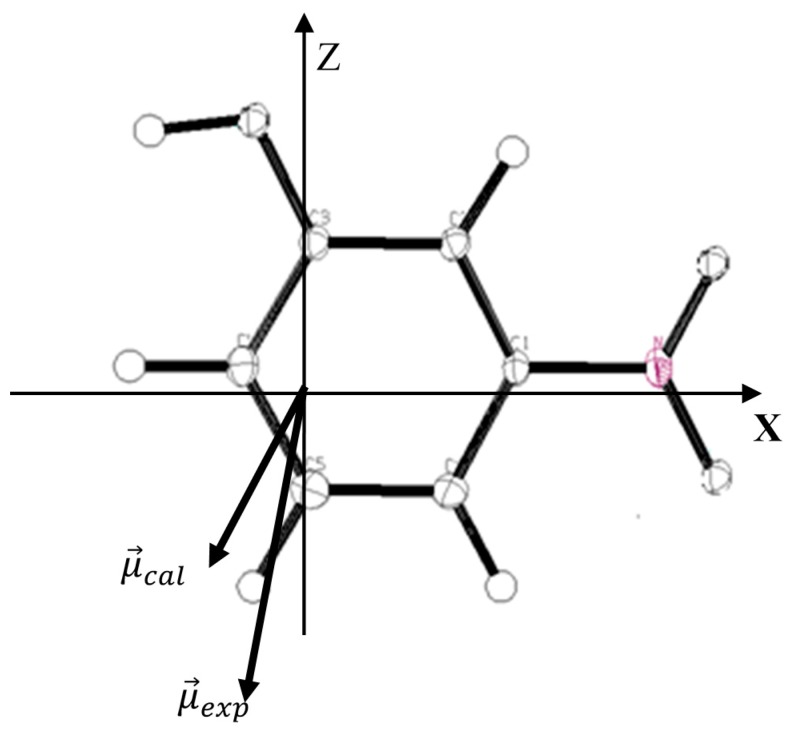
Orientation of the molecular dipole moment of m-NPH: ${\vec{\mu }}_{\exp}$: molecular dipole moment from the experimental study; ${\vec{\mu }}_{cal}$: molecular dipole moment from the theoretical DFT calculations.

If $\Delta \rho \left(\vec{r}\right)$ rather than $\rho \left(\vec{r}\right)$ is being considered the electrostatic moment due to the deformation density in the molecule and can be estimated. The experimental molecular dipole moment of m-NPH has been determined in the previous paper cited above using the multipolar model [5]. Such studies have clearly evidenced the electron donor character of the C-H groups in conjunction with the electron acceptor character of the nitro and hydroxyl groups. In general, the experimental method provides a magnitude of about 5.80 Debye for the dipole moment. A theoretical calculation has been performed usingB3LYP at 6-31G* basis set in order to carried out the components of the molecular dipole moment. The obtained results are summarized in Table 6 in which the experimental values are given for comparison. The orientation of the different vectors of dipole moment in the molecular axial system is shown in Figure 5.

**Table 6:** Components of the molecular dipolar moment from DFT calculations (B3LYP at 6-31G* basis set) and X-ray experiment. The origin coincides with the center of mass of the molecule, and the Cartesian system referred to the inertial axis of the molecule.

Methods	Models	*μ_X_*	*μ_Y_*	*μ_Z_*	*μ* Debye
X-ray Experiment	Multipolar refinement	−0.3209	−0.3200	−6.3358	5.8000
*Ab initio*	DFT(B3LYP/6-31G*)	−2.1194	−0.0010	−5.4234	5.8228

The components of the electrostatic quadrupole moment are obtained by substituting in Equation (3) the operator *Ô(r)* by ${\vec{r}}_{\alpha }\Lambda {\vec{r}}_{\beta }$. If in that equation the density function $\rho \left(\vec{r}\right)$ is replaced by the multipolar expansion up to order *l* = 1, then the components of the quadrupole moment are given by:

(4) $$Q_{\alpha \beta }=\sum \left[Q_{\alpha \beta }^{i}+r_{i\beta }d_{i\alpha }+r_{i\alpha }d_{i\beta }+r_{i\alpha }d_{i\beta }q_{i}\right]$$

where *d_iα_* and *q_i_* represent respectively the component of the dipole moment and the net charge of atom i at *r_i_*. $Q_{\alpha \beta }^{i}$ are the atomic quadrupoles neglected here.

In the case of the direct integration method the development of Equation (3) leads to:

(5) $$Q_{\alpha \beta }=\frac{1}{V}\sum_{\vec{H}}\Delta F\left(\vec{H}\right)\left[\sum_{i}\left(Q_{\alpha \beta }^{i}+r_{i\beta }d_{i\alpha }+r_{i\alpha }d_{i\beta }+r_{i\alpha }d_{i\beta }q_{i}\right)\right]$$

with:

(6) $$Q_{\alpha \beta }^{i}=\int_{t_{i}}\left(r_{\alpha }-r_{i}\right)\left(r_{\beta }-r_{i}\right)e^{i2\pi \vec{H}\left(\vec{r}-{\vec{r}}_{i}\right)}d^{3}r$$

The summation over $\vec{H}$ is performed over all structure factors and the indice *t_i_* designates the integrable subunits. Evaluation of all molecular moments requires summations of the density and moments of each subunit which are being performed according to a space partitioning scheme. The quadrupolar moment values are reported in the Table 7 with the analogous components obtained from the point charge model using the net atomic charges derived by NPA method calculations. The most remarkable features when comparing experimental values with those derived from the free molecule stand-out in the *Q_XX_*, *Q_ZZ_* and *Q_XX_* components. The experimental second moment component relative to a chosen molecular origin, (*Q_XX_* = −55.53, *Q_ZZ_* = −63.88) shows a weaker charge expansion than in the free molecule (*Q_XX_* = −53.63, *Q_ZZ_* = −51.53) while the positive *Q_ZZ_*’s indicate a similar contraction in the $\left(\vec{X}+\vec{Z}\right)$ direction (orientations in the molecular frame given in Figure 5) for both the free molecule and the molecule in the crystal state. On the other hand the same electronic delocalization in the $\left(\vec{X}+\vec{Z}\right)$ direction is being observed in the molecular plane for molecules in both states. 

**Table 7:** Components of the molecular quadrupole moment of the charge distribution (e.Å²) from theoretical calculations and experimental electron density study.

Quadrupole Moments	X-ray Experiment	*Ab Initio* DFT(6-31G)
*Q_XX_*	−55.532	−53.632
*Q_YY_*	−53.129	−53.777
*Q_ZZ_*	−63.886	−51.536
*Q_XY_*	−1.825	0.964
*Q_XZ_*	3.878	0.002
*Q_YZ_*	−1.755	−0.001

### 4.5. Frontier Molecular Orbital Analysis

Molecular orbitals (HOMO-LUMO) and their properties such as energy are very useful for physicist and chemists and are very important parameters for quantum chemistry. This is also used by the frontier electron density for predicting the most reactive position in π-electron systems and also explains several types of reaction in conjugated system [19]. The conjugated molecules are characterized by a small highest occupied molecular orbital- lowest unoccupied molecular orbital (HOMO-LUMO) separation. Both the highest occupied molecular orbital and lowest unoccupied molecular orbital are the main orbitals which take part in chemical stability. The HOMO represents the ability to donate an electron, LUMO as an electron acceptor, represents the ability to obtain an electron. The HOMO and LUMO energy calculated by B3LYP/6-311++G(d,p) method is shown below. This electronic absorption corresponds to the transition from the ground to the first excited state and is mainly described by one electron excitation from the highest occupied molecular orbital to the lowest unoccupied molecular orbital. While the energy of the HOMO describe the ionization potential, LUMO energy is concerned by the electron affinity Energy difference between HOMO and LUMO orbital is called as energy gap which is an important stability for structures and is calculated as:

HOMO energy =−0.264 au
LUMO energy =−0.106 au
HOMO-LUMO energy gap =−0.158 au

It has been shown that calculated energy gap between HOMO and LUMO can be very useful to prove the activity from intramolecular charge transfer [20].

### 4.6. Electrostatic Potential 

In order to grasp the molecular interactions, the molecular electrostatic potential (MEP) is used. The molecular electrostatic potential is the potential that a unit positive charge would experience at any point surrounding the molecule due to the electron density distribution in the molecule. The electrostatic potential is considered predictive of chemical reactivity because regions of negative potential are expected to be sites of protonation and nucleophilic attack, while regions of positive potential may indicate electrophilic sites.The distribution of the electrostatic potential for the molecule in the crystal was calculated from Equation (7):

(7) $$\Phi \left(r\right)=\int \frac{\rho_{total}\left(r\right)}{\left|r-r\prime \right|}dr$$

where $\rho_{total}$ represents both the nuclear and the electronic charge. The integration is over the molecular volume, and r′ represents the atomic position relative to same origin. The integration includes the atoms of only one molecule and therefore does not include directly the effects of charge distribution of the molecules. 

**Figure 6:**
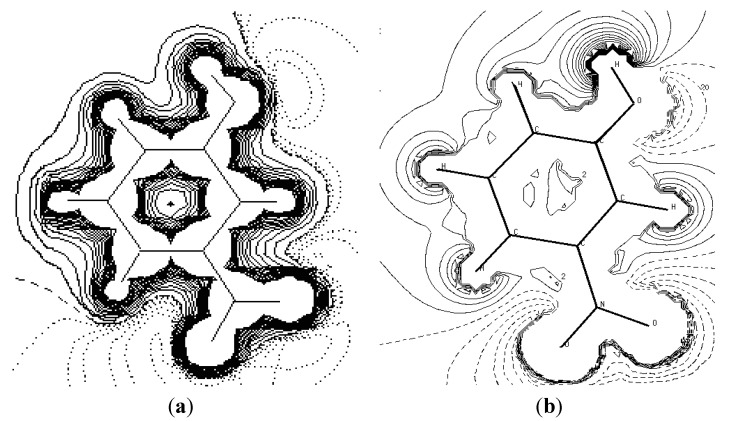
The electrostatic potential maps around the molecule. The section is in the plane of the ring atoms. (**a**) Experimental (contours are at 0.05 eǺ^−1^). (**b**) Theoreticalusing the Density Functional Theory at B3LYP level of theory at 6-31G* (contours are at 0.025 eǺ^−1^). Zero and negative contours are dashed lines (1 eǺ^−1^ = 332.1 kcal·mol^−1^).

**Figure 7:**
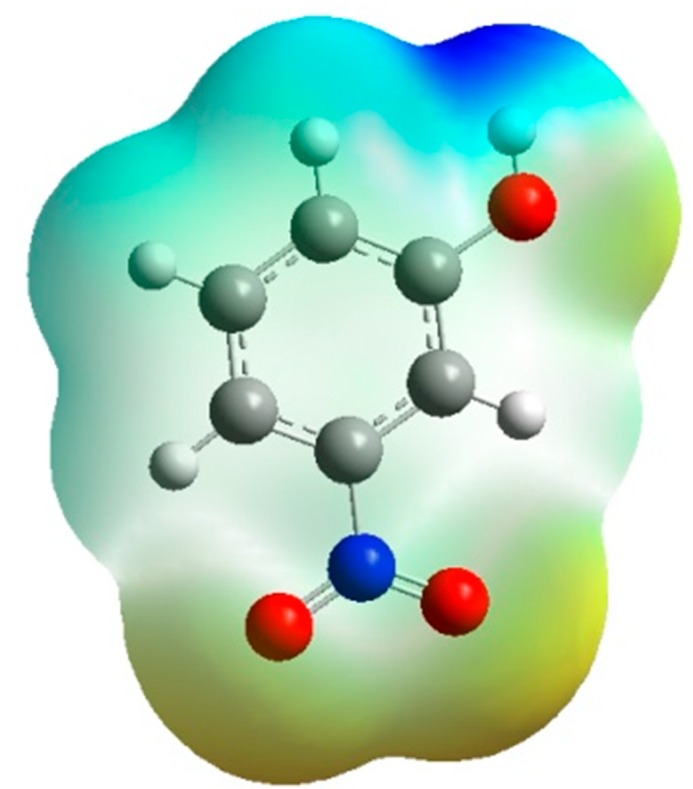
3D-representation of the electrostatic potential around the molecule using the Density Functional Theory at B3LYP level of theory at 6-31G*

Figure 6 shows the experiment and theoretical maps of the electrostatic potential distribution in the plane of the base ring. We are used the Density Functional Theory at B3LYP level of theory at 6-31G* to describe the theoretical electrostatic potential map. Figure 7 is the same representation in 3D dimensions of the theoretical electrostatic potential map. The extension of the positive electrostatic potential around the C-H group and the regions of negative electrostatic potential around the nitro and hydroxyl group gives same conclusion about the nature of the intramolecular charge transfer as found by the orientation of the molecular dipole moment.

The potential of the *m*-nitrophenol molecule has been calculated from the experimental electron density distribution by the multipolar method using the X-ray diffraction data. The comparison of the experimental potential in a crystal and the theoretical potential for an isolated molecule is an excellent test for high quality descriptive model for the electron charge density distribution from X-ray diffraction experiment.

## 5. Conclusions

In this article, we have dealt with the salient features of the electronic charge density distribution in molecular solids obtained by both theory and experiment. This study has obtained good accurate results on the structure and electron charge density which back the experimental results for the electron charge density distribution.

The general conclusion from the estimation of the dipolar moments and the electrostatic potential of the *m*-nitrophenol molecule in the both experimental and theoretical study is that the region of the nitro and hydroxyl groups is electronegative and the C-H group region is electropositive. These results could be used to explain the existence of the polymorphism in *m*-nitrophenol compounds, if they were completed by the study of the nature and the energy of the molecular interaction by the X-ray diffraction of the both polymorphic of m-NPH.
